# One-click device for rapid visualization and extraction of latent evidence through multi-moding light source integration and light-guiding technology

**DOI:** 10.1038/s41598-022-21136-0

**Published:** 2022-10-10

**Authors:** Xuejun Zhao, Nengbin Cai, Xiaochun Huang, Wenbin Liu, Fei Gao, Changliang Wang

**Affiliations:** 1 Shanghai Key Laboratory of Crime Scene Evidence, Shanghai Research Institute of Criminal Science and Technology, Shanghai, 200083 China; 2 Shanghai Police College, Shanghai, 200137 China; 3 Shanghai Key Laboratory of Crime Scene Evidence, Shanghai Institute of Forensic Science, Public Security Bureau, Shanghai, 200083 China; 4 Shanghai Public Security Bureau, Shanghai, 200040 China

**Keywords:** Optics and photonics, Applied optics, Lasers, LEDs and light sources

## Abstract

Visualizing latent evidence at a crime scene has gained popularity in the field of forensic science during the past few years. Thus, this study designs and develops a one-click device for the rapid visualization and extraction of latent evidence through multimodal light source integration and light-guiding technology. Our device exhibits multispectral and angle timing functions for storing the captured evidence images. Furthermore, the geometric registration, feature extraction, feature optimization, and feature integration of the evidence images are processed by a backend system, and the images are then presented. Overall, this study enhances the standard and the technical content of evidence extraction and simplifies the evidence extraction process. In addition to the rapid handling of the scenes captured at a crime scene, the one-click device has other notable advantages, such as fast imaging, portability, being independent of the environmental conditions and the operator’s technical capabilities, and zero pollution to ensure the repeatability of material evidence extraction. Compared with the original optical forensics equipment, the spectrum and angle of our system are more extensive.

## Introduction

Crime scene evidence collection methods are primarily divided into display and extraction. The former methods mainly include a physical display (including optical display) and a chemical display^1,2^, while the latter methods involve original and photo extraction. In recent years, due to the rapid development of new technologies, such as DeoxyriboNucleic Acid (DNA) testing^1–6^ and non-destructive displaying methods, optical display methods have gradually become the mainstream^7–9^, with photography governing the extraction methods. Nevertheless, the above methods have a wide variety and involve cumbersome operations, heavily relying on the personnel’s technical level, often affecting the quality of evidence collection^10,11^. For instance, during optical display, the quality of evidence collection is affected by numerous parameters such as the selected spectrum, light distribution angle, photography technology, and the selected filter^12–14^. In order to further improve the crime scene investigation ability, assist the case investigation department in dealing with the scene quickly in case of emergencies, and ensure the quality of crime scene evidence collection, it is mandatory to develop an easy-to-use and fully automatic device for rapid visualization and extraction of latent evidence.

## Literature review

Crime scene evidence collection typically aims to detect and extract biological stains to support downstream processing of forensic samples and generate rapid intelligence to assist the investigation department in identifying the person the biological stain belongs to. Current approaches traditionally rely on chemical and immunological techniques, which lack sensitivity and specificity. Furthermore, existing evidence collection techniques are primarily manual, opposing the current era of automation that governs various domains in everyday life.

The oldest forms of biological forensic analysis rely on fingerprints or body fluids, e.g., saliva, blood, and semen, detected at a crime scene^15^. Alternative methods exploit DNA profiling^16^ and detect the gunshot residue on an object or even the detected footprints. Nevertheless, most of these techniques are manually applied, increasing the burden of the crime scene evidence collection team, and are significantly governed by the operator’s technical abilities. Spurred by this concern, the “Future of Forensic and Crime Scene Science Conference” defined the critical system and technological design drivers^17^ as (1) Miniaturization to increase portability and ease of use, (2) Faster analysis, (3) Simple ‘Black-Box’ interpretation, (4) Easy integration of case information, and 5. Low cost. Urged by these developing drivers, the iForenLIBS^18^ device has been developed to collect Gunshot Residue (GSR) from a crime scene, which exploits Laser Induced Breakdown Spectroscopy (LIBS) technology to analyze GSR particles by detecting the three typical ammunition elements (Lead, Barium, and Antimony) simultaneously. An alternative method^19^ analyzed GSR by employing a chemical imaging system operating in the short-wave infrared region (SWIR). Although this hardware configuration did not meet the portability design driver mentioned above, it still poses an appealing solution for automatic crime evidence collection.

It should be noted that according to the literature, currently, these are the only devices developed at a Technology Readiness Level (TRL)^20^ of at least 6, where the device undergoes some field tests while still considering (at least partially) the proposed design drivers mentioned above^17^. However, several appealing approaches exist that are still at a lower TRL of up to 3 or 4. Indeed, the great success of modern computer vision techniques, including deep learning strategies, has already been extended to crime scene evidence collection or supporting crime investigation teams in general. For example, in a footwear print retrieval system^21^, the footwear print pattern is characterized based on shape features and an Attributed Relational Graph. Recently, 3D crime scene reconstruction^22^ technology has been developed, producing more insightful, complete, and objective documentation for crime scenes. This system comprises a laser scanner, a situational structured light scanner for fine measurements, and a detailed structured light scanner to extract the maximum possible details.

Currently, fingerprint and body fluid stain collection are manual, and the investigation scene's surface must be considered to determine the appropriate collection methods. Depending on the surface type, i.e., absorptive, non-absorptive smooth surface, and non-absorptive rough surface, the appropriate powder type for fingerprint extraction is chosen. Furthermore, the collecting methods depend on the fingerprint type, i.e., latent, patent, and plastic^23^. For instance, to collect latent fingerprints made from body skin oil and sweat, visualizing the fingerprint requires an additional process^24^. Therefore, given the current technological era and to ease the crime investigation departments, developing a fully autonomous, i.e., one-click, device to capture fingerprints is essential. Nevertheless, to our knowledge, such an automatic fingerprint extraction system that meets the required design drivers^17^ and exceeds a TRL level of 4, i.e., exceeding the proof-of-concept stage, has not been proposed yet.

For completeness, opposing fingerprint and body stain fluids visualization and extraction, most current work focuses on automatically classifying and matching the collected evidence against a database rather than collecting them. Hence, significant improvements have been made at a software level. For instance, fingerprint classification, i.e., grouping fingerprints in a consistent way so that different impressions of the same finger are clustered in the same group, relies on deep learning^25–29^, graph theory exploiting directional data^30^, ridge flow and ridge lines^31,32^, and rule-based^33^ schemes. Nevertheless, these techniques are purely software-based and are the follow-up process of this paper’s visualization and extraction process.

Spurred by this technology gap, this paper develops a “one-click device for rapid visualization and extraction of latent evidence”, aiming to develop and evaluate a fully autonomous, easy-to-use, quick, and highly efficient fingerprint and body fluid stains extraction system (image capturing) for crime scene evidence collection in the crime field scene. The proposed device improves the scientific and technological content of crime scene investigations, simplifies the evidence collection steps, and quickly completes the display and extraction of latent evidence, such as fingerprints and body fluid stains from the scene, based on a one-step process. This is important as our system neglects the impact of cumbersome operation steps and the personnel’s technical level on the quality of forensics, ensuring standardization, efficiency, and high-quality crime scene evidence collection, overcoming the deficiencies of existing methods. According to the user reports of several front-line investigation departments in Shanghai, the developed device acquired multi-angle fingerprint photos on non-plane bearing objects at the scene of many criminal cases. After image fusion, clear and complete fingerprint pictures were obtained. Thus, the proposed device provides a robust technical guarantee for the public security teams to solve criminal cases. Overall, the contributions of this work are summarized as follows:

1. Exploiting the optical properties of biological evidence and its carriers, employing the mechanism and technology of various light source bands to display biological evidence, and integrating multiple light sources and image processing techniques.
2. Developing a “one-click device for rapid visualization and extraction of latent evidence at the scene” by employing the knowledge presented above. The developed system is portable with a high battery endurance, meeting the suggested design drivers for the crime evidence collection systems.
3. Solving the current problem of rapid search and discovery of potential biological evidence within a crime scene while avoiding complicated operation procedures, eliminating the effect of different personnel's technical skills on the quality of the collected evidence, and meeting the requirement for rapid disposal of crime scene evidence.
4. Through the proposed one-click device, our system acquires multiple photographs with multispectral and multi-angle lights, while the evidence’s geometric registration, feature extraction, optimization, and fusion are completed in the background before the resultant image is displayed.
5. Although the developed device has numerous capabilities, it is a low-cost device as its manufacturing cost does not exceed $1200.

The remainder of this paper is as follows. In “Literature review” section presents the current works in crime scene evidence collection. In “Study on multi-mode light source integration and light-guiding technology” section introduces the multi-mode light source integration and light-guiding technology, while “Design of the proposed device” section presents the design of the developed one-click device for rapid visualization and extraction of latent evidence at a crime scene. Finally, “Conclusion” section concludes this work.

## Study on multi-mode light source integration and light-guiding technology

A one-click device for rapid visualization and extraction of latent evidence at the crime scene should be portable and have the endurance of self-power supply for a long time. Therefore, this project mainly focuses on developing light-emitting diode (LED) light source technology and studies the multi-integral spherical focusing technology. An integrating sphere is a hollow sphere whose inner wall is coated with white diffuse reflective material, also known as a photometric sphere or luminous flux sphere. One or several window holes are opened on the spherical wall used as light inlet and receiving holes for placing the light receiving devices. The inner wall of the integrating sphere should be a highly accurate sphere that does not deviate from the ideal sphere of more than 0.2% of the inner diameter. The inner wall of the ball is coated with ideal diffuse reflection material, i.e., a material with a diffuse reflection coefficient close to one. Due to the “integration” effect of the diffuse reflection coating on the inner wall of the integrating sphere, in theory, uniform Lambert illumination is obtained at any position on the light outlet plane of the integrating sphere, and the number of lights can adjust the brightness level, and the color temperature is unchanged. Thus, spurred by the advantages of multi-integral spherical focusing technology, this paper designs an integrated spherical diffuse reflection light source that integrates the primary encapsulation and secondary optical design of LED light source, simplifies the light source structure, and improves luminous efficiency.

**Figure 1:**
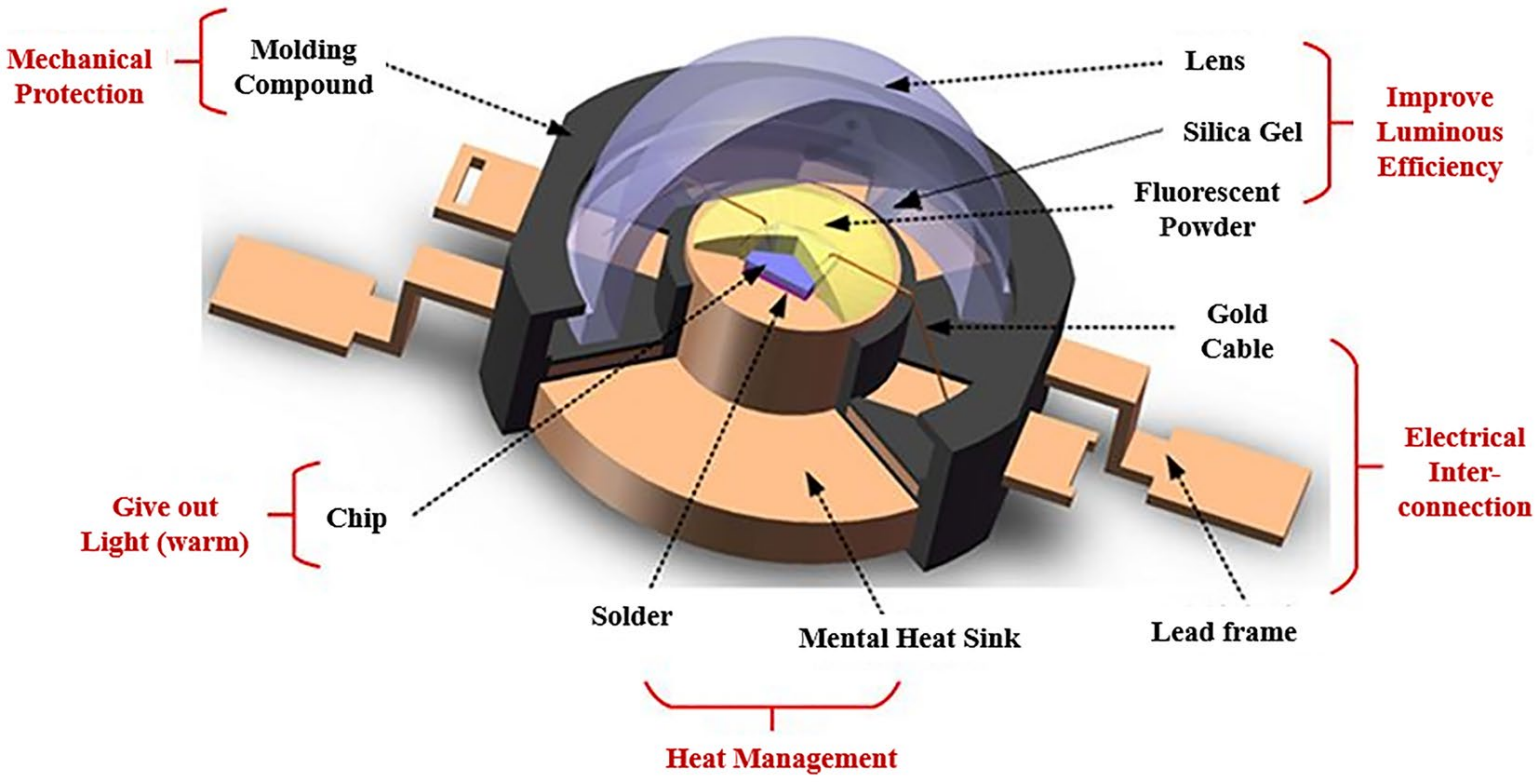
Integrated primary encapsulation and secondary optical design of the LED light source.

The primary encapsulation of the device adopts high directivity LED encapsulation technology, utilizes pointed epoxy encapsulation, and does not involve a scattering agent. Additionally, the half value angle is 5°–20°, affording the light to converge. Simultaneously, the space between the chip and the lens is filled with silica gel to encapsulate and fix the electrode and lead. As illustrated in Fig. 1, the secondary optical design transforms the lens into a multi-sphere area ball by combining the LED chip with the base and the multi-sphere encapsulated lens. The LED chip is a surface light source with a front surface of 1 × 1 mm, and the distribution angle of the light field is 180°, dividing the light field distributed by the LED nearly 180° into two parts: the small angle divergent light cone in the center and the large angle light at the edge. In order to ensure that both kinds of light improve the light collection efficiency based on the light’s uniform transition at the junction, both lights must be converted into a nearly collimated light through different optical paths in the lens. Therefore, first, the loss and refractive indexes are calculated using the principle of engineering approximation, where the surface shape is determined by weighting the two edge rays of the LED surface light source, and the light passes through the geometric lens center. Then, to balance the optical parameters such as light extraction efficiency and divergence angle, three-dimensional simulation modeling, light tracing, continuous adjustment, and repeated optimizations are performed by changing the arrangement and combination of the multi-spherical LED encapsulation lenses. This strategy affords to find the most effective near collimated illumination beam and realize the illumination output with an extensive dynamic range and stable color temperature.

## Design of the proposed device

**Figure 2:**
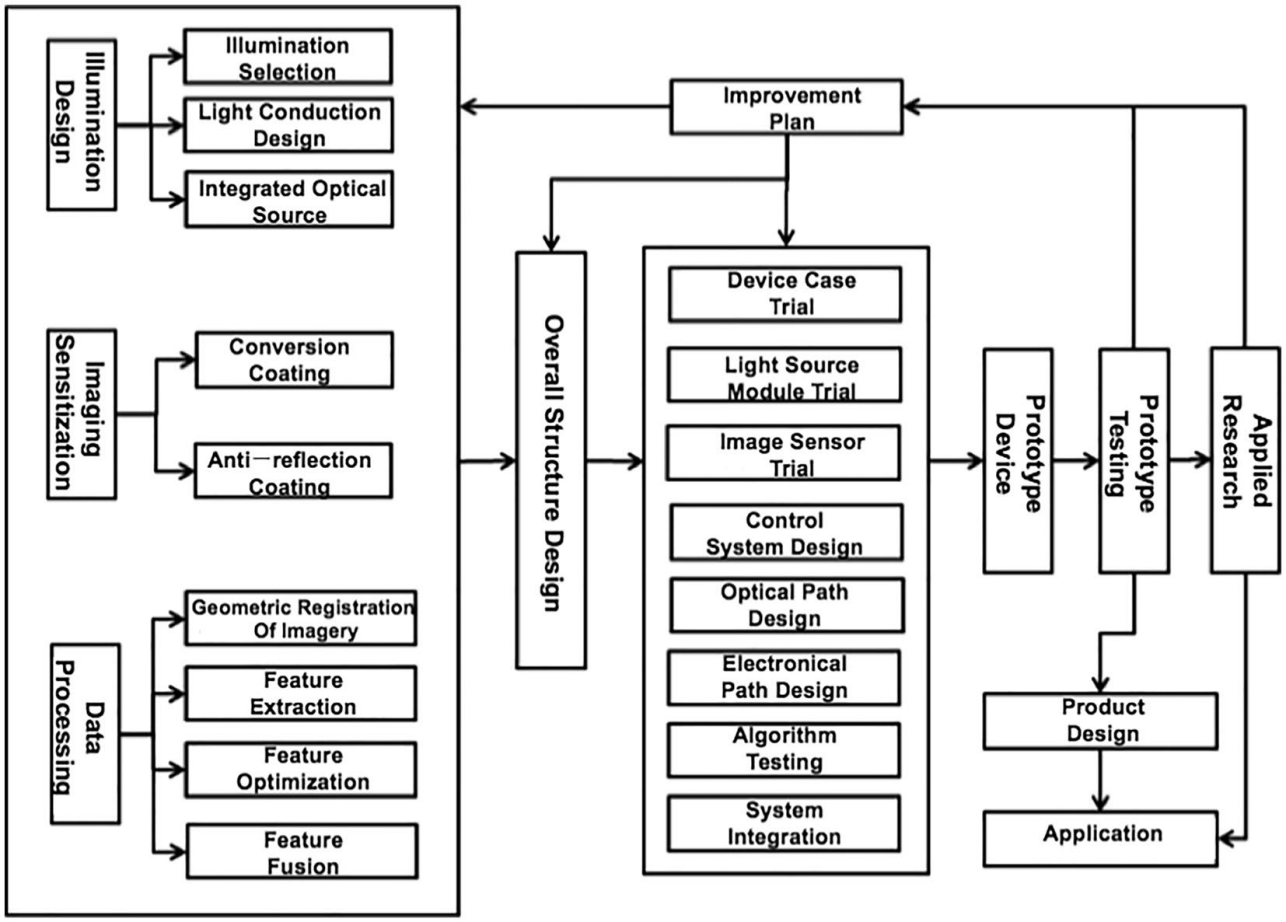
General technical route of the "one-click device for rapid visualization and extraction of latent evidence at the scene".

The overall objective of this study is to develop a “one-click device for rapid visualization and extraction of latent evidence at the scene” of TRL 6. Precisely, the device visualizes and extracts latent evidence at a crime scene with one click, i.e., photographs of the physical evidence are acquired with multispectral and multi-angle lights using a one-click process, whose geometric registration, feature extraction, feature optimization, and feature fusion, are completed in the background before the resultant image is displayed. The technical design route of the “one-click device for rapid visualization and extraction of latent evidence at the scene” is illustrated in Fig. 2. According to this study’s overall goal, the developed device meets the requirements of lossless display of potential traces at the scene by utilizing the following optical methods to fulfill this goal. First, we study the multi-model light source integration and light-guiding technology, the imaging device’s sensitization technology, and the image feature optimization algorithm. Subsequently, the proposed device's overall structural design and system integration are carried out based on the study's outcomes. Following this stage, the prototype trial manufacturing, tests, and improvements, as well as applied research and product design, are performed.

The device’s hardware mainly comprises a light source, imaging, and control system. Regarding the control system, a tablet computer type is embedded, utilizing integrated CPU and GPU, which typically have a medium-level configuration such as Qualcomm snapdragon 870 and 8 GB of memory.

**Figure 3:**
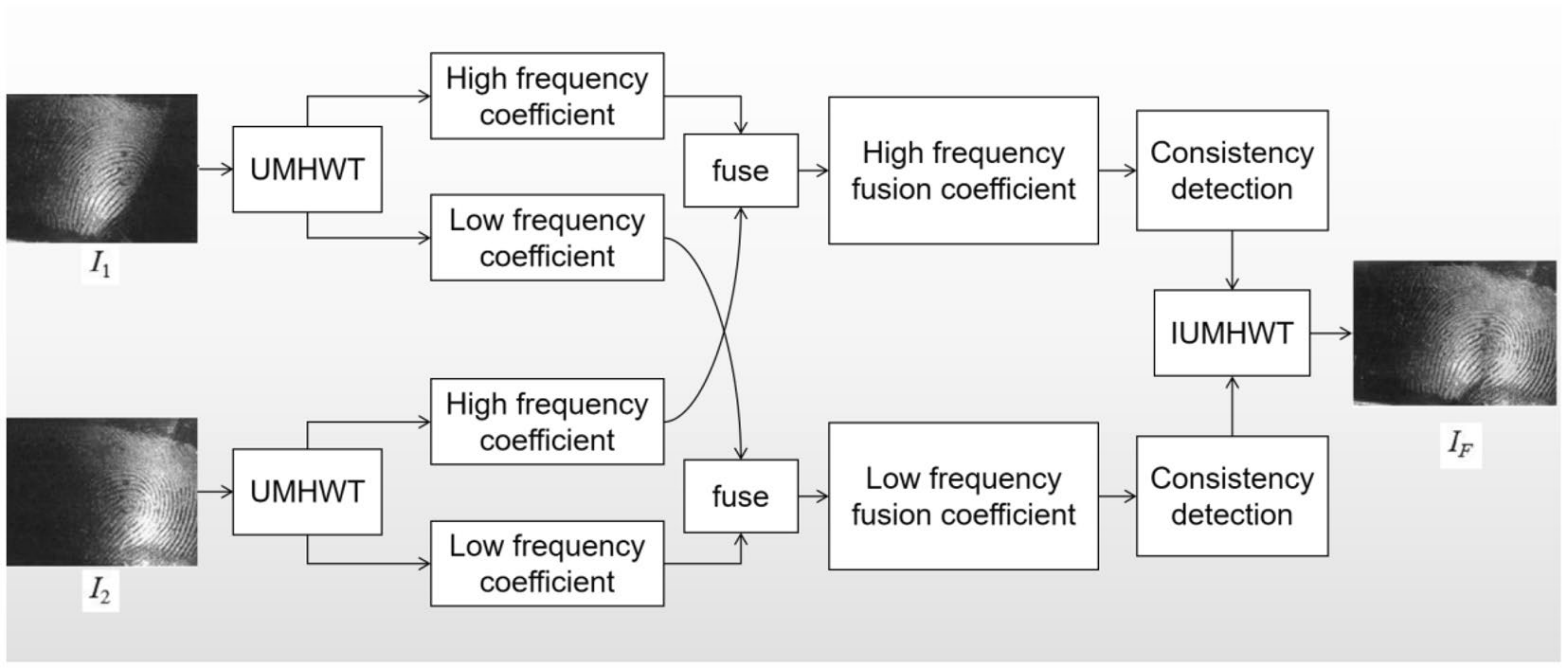
Flow chart of background image processing steps.

Figure 3 depicts that the device’s algorithm must complete the geometric registration, feature extraction, feature optimization, and feature fusion of the physical evidence image set. Specifically, first, the Undecimated Morphological Haar Wavelet Transform (UMHWT) is applied to the source images I1 and I2 to obtain the low-frequency coefficients, i.e., decomposition coefficients on the total scale J (the decomposition layers of UMHWT) and the high-frequency coefficients, scale function coefficients on each scale J (0 < J ≤ J). The low-frequency and the high-frequency coefficients are fused according to the corresponding fusion rules (low and high, respectively), then both frequency fusion coefficients undergo a consistency verification process, which is finally updated utilizing the detection results. The final low-frequency and high-frequency fusion coefficients are inversed using the inverse UMHWT (IUMHWT) to obtain the final image fusion (IF).

**Figure 4:**
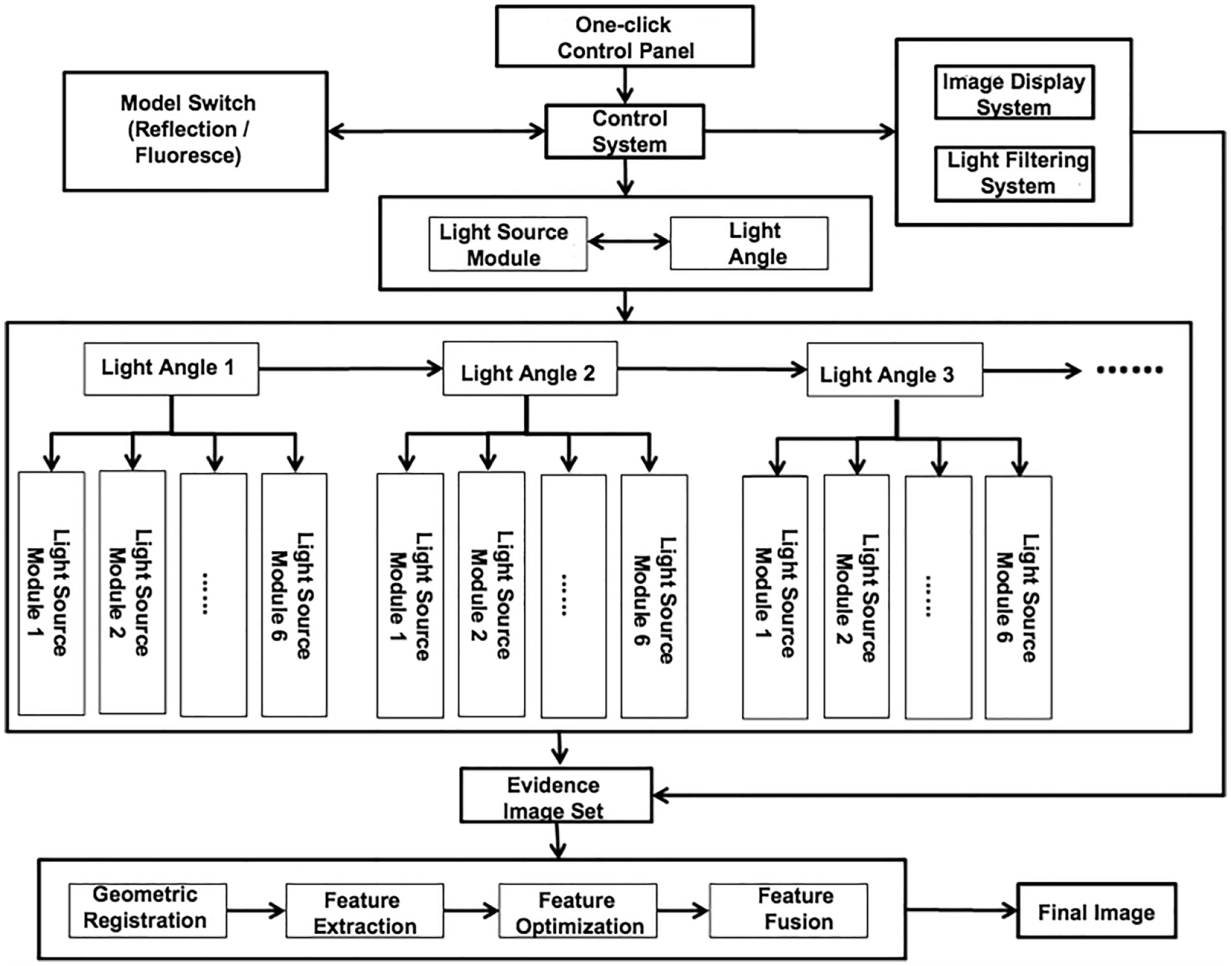
Functional design of the one-click control system.

Figure 4 illustrates the functional design of the device’s “one-click” control system. The one-click control panel utilizes a high-definition LCD touch screen with a high-definition display and an intelligent one-button operation function. The control system is the core of the intelligent one-key operation. The optical, mechanical, and electrical control programs, image registration, feature optimization, and image fusion algorithms are embedded in the control system’s chip, which is responsible for the automatic control mode switching (the system involves two optical schemes of reflection and fluorescence), automatically switches the light source, and performs automatic filtering and imaging.

**Figure 5:**
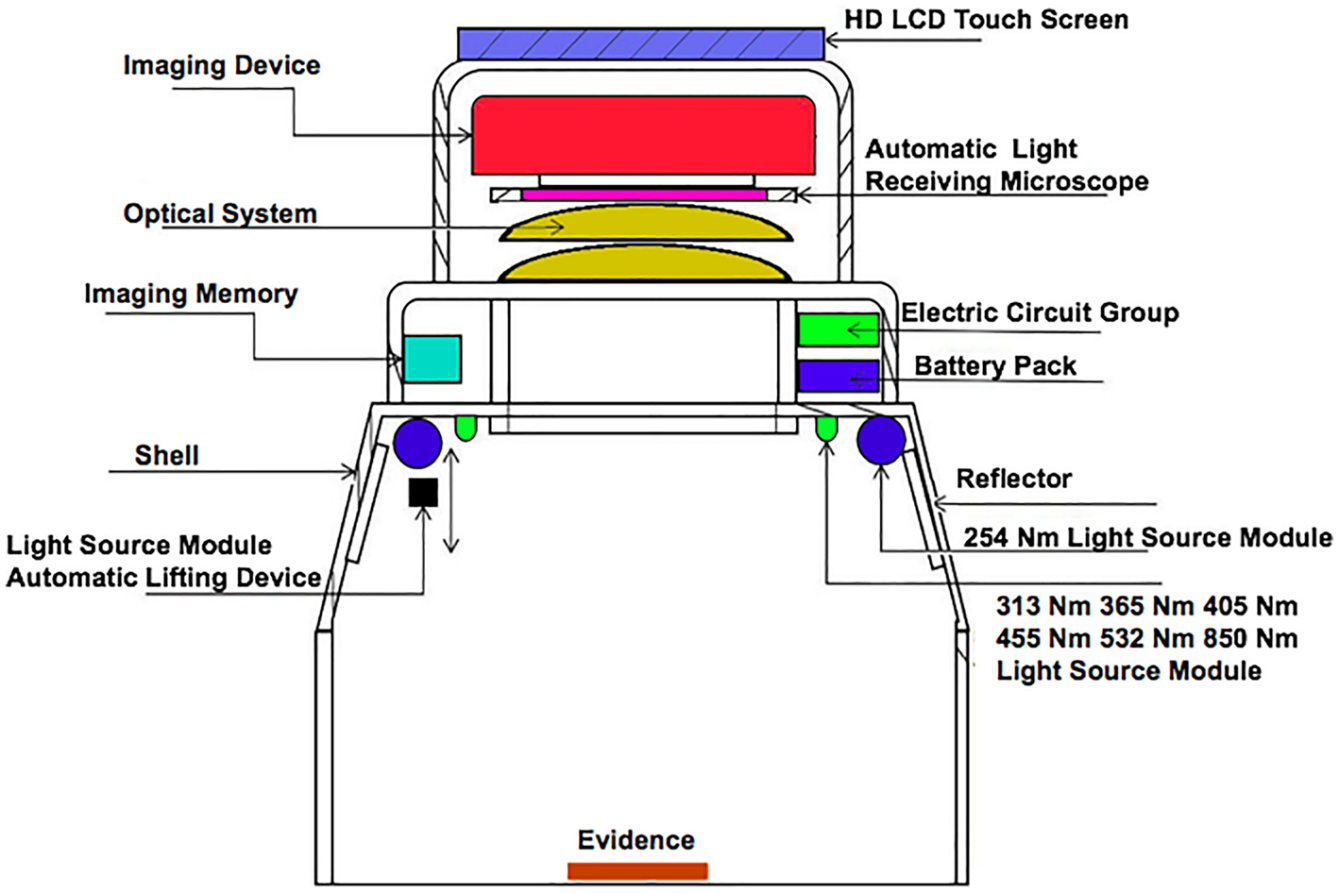
The overall design of the equipment.

**Figure 6:**
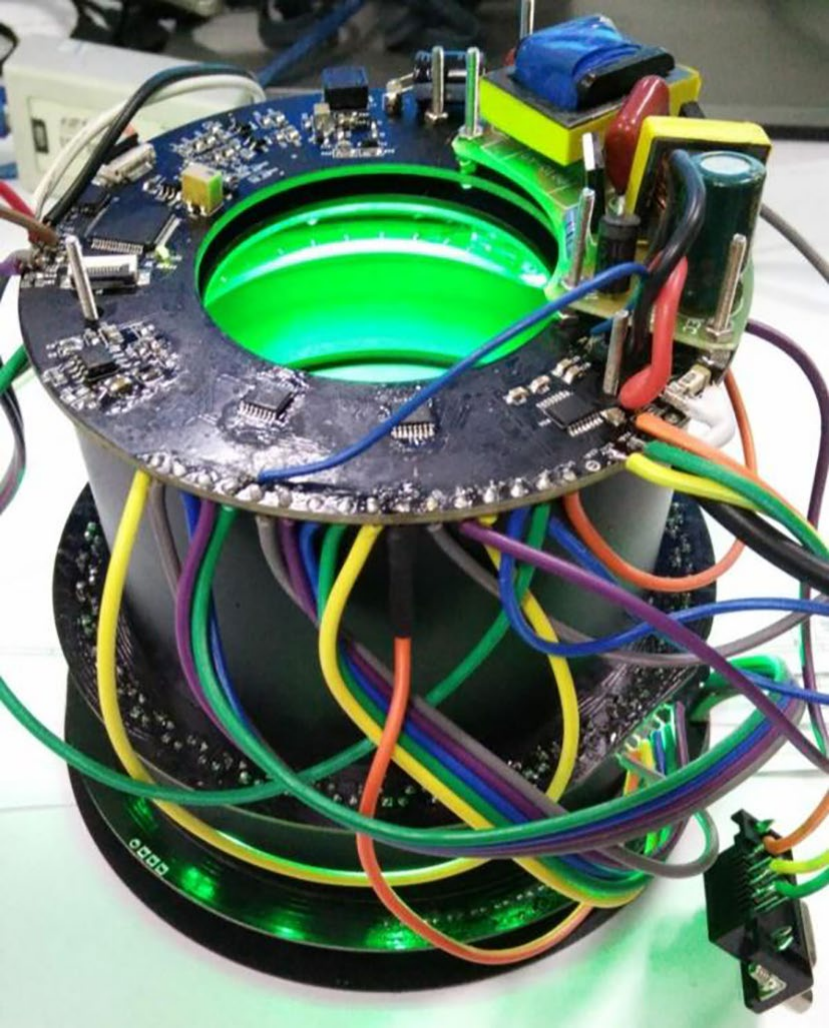
Physical diagram of components.

**Figure 7:**
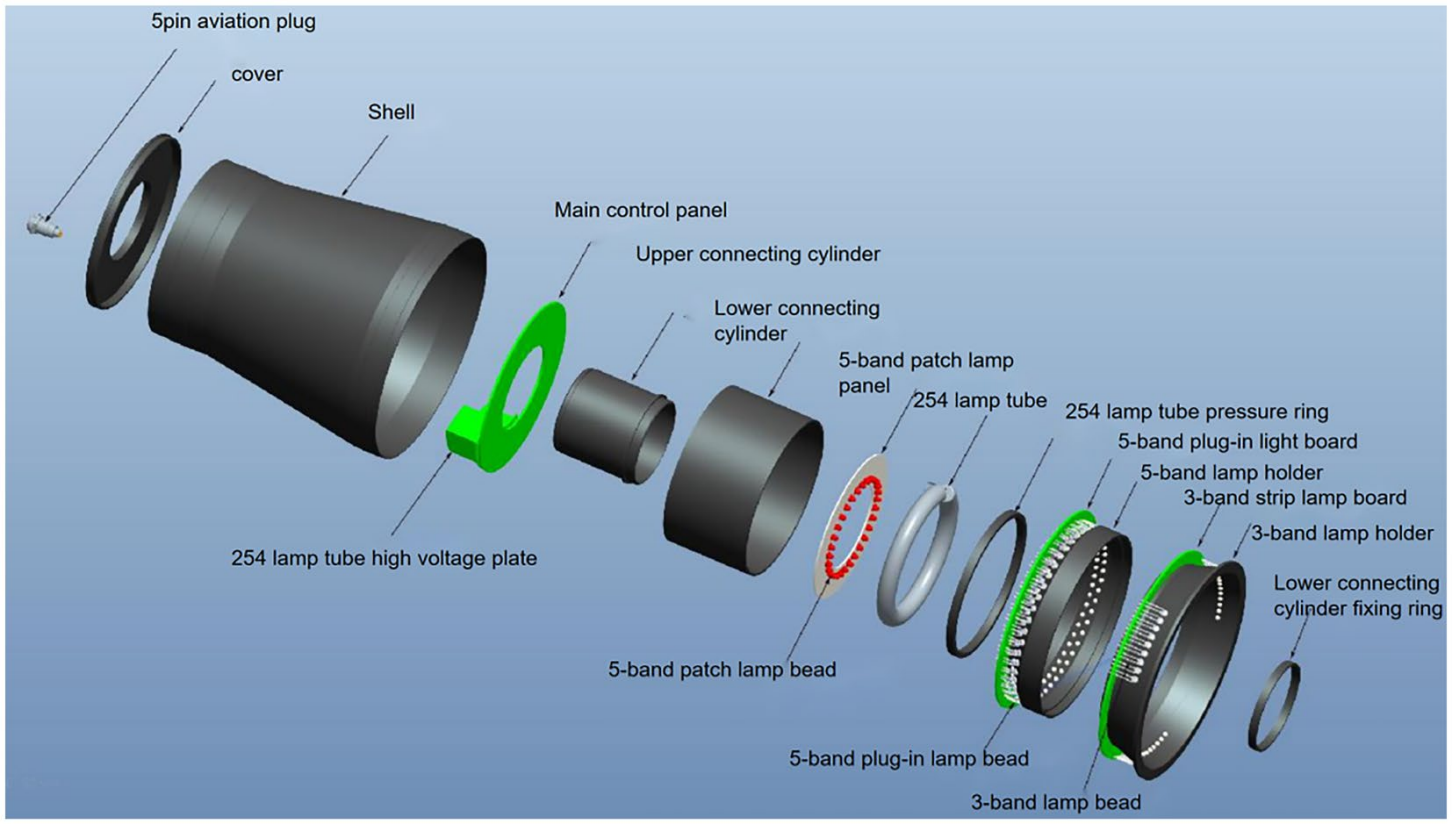
The configuration diagram of the equipment.

**Figure 8:**
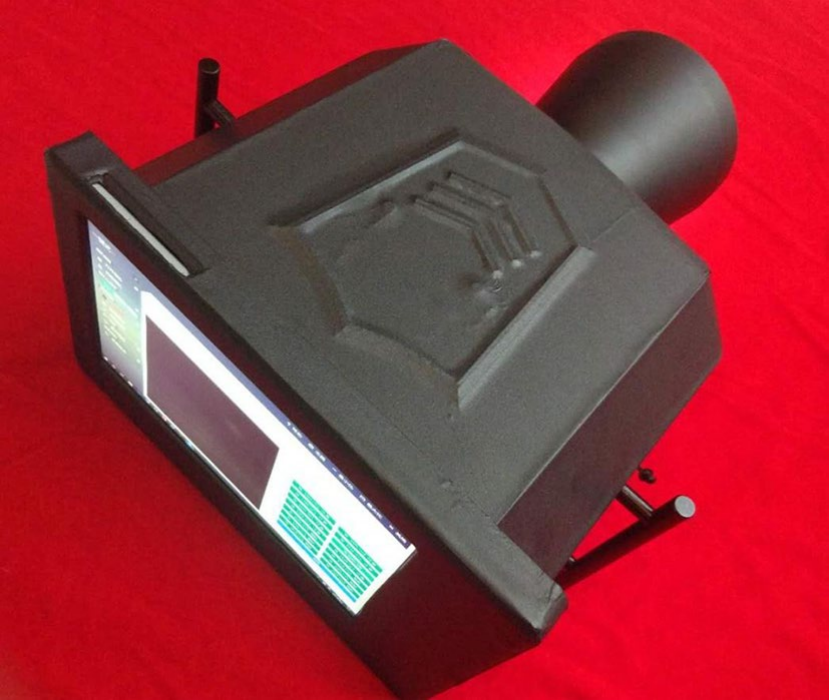
Physical diagram of the equipment.

**Figure 9:**
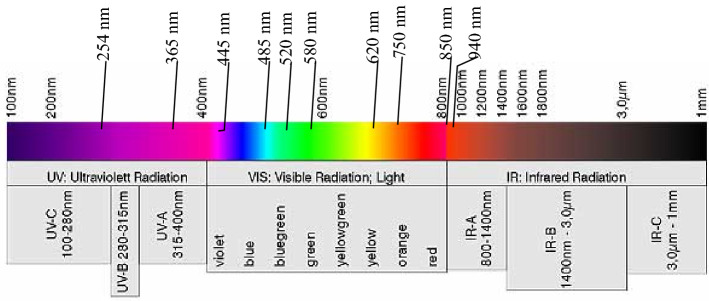
Spectral characteristics of the device’s light source^34^.

Figure 5 depicts the overall equipment design, with the related system components presented in Fig. 6. Figures 7 and 8 illustrate the device’s configuration and physical diagram. According to the test report of the Shanghai Institute of quality inspection and technical research, the entire device, including a battery weight of 4.3 kg, comprises six parts and has the following operations:

1. Camera control. This includes two functions: turning on and off the camera. The operator clicks the button to turn the camera on, and the camera will start outputting images. The operator clicks the button to turn the camera off, and the camera stops outputting images.
2. Camera resolution. Selecting the 1024b indicates that 2 × 2 pixels are combined to produce a map of a 1024 × 1024 pixels resolution. 1024c indicates the window’s center that can be used for drawing, where the image resolution is 1024 × 1024 pixels. Finally, selecting 2048, the operator can plot on the image of resolution 2048 × 2048 pixels.
3. Camera control involves autofocus and automatically or manually acquiring the photograph. In the autofocus mode, the instrument calibrates different wavelengths and filters. If this option is selected, the camera is automatically adjusted to the calibrated focus position during data acquisition, i.e., automatic or manual photography, while the previous user-defined focusing operation is discarded. When the automatic photograph acquisition (one click acquisition) is selected, the parameter setting box appears on the screen. Then, by simply clicking the “acquisition” button, the system automatically acquires the pictures after combining 14 light sources with different wavelengths and three filters (14 × 3 = 42 images in total). The user can select the filter and light source of interest during automatic photographing through the drop-down list. Finally, when manual photographing is selected, the parameter setting box appears on the screen. According to the characteristics of the tested material and the operation tips, the user manually selects the filter and light source and observes the effect of parameter combination in the “real-time image display area”. When the effect is the best, the operator clicks the “acquisition” button, and the system will collect the image data under the parameter setting.
4. Image fusion. This function is used to fuse multiple images through the built-in algorithm. The user clicks the “image fusion” option, and the screen presents the images. Then, the user selects the pictures to be fused (more than two), clicks the “image fusion” button on the interface, and the system automatically fuses the selected pictures. After the operation is completed, the “processed” picture will appear on the screen.
5. Filter switching is used to select the filter applied. The operator clicks the “filter switch button to open or close the filter switch menu, providing the following options. Empty filter, where the device does not use any filter, 550 filter, to switch to the 550 nm filter environment, and 580 filter, to switch to the 580 nm filter environment.
6. Light source switching. This option selects the light source. The user clicks the “light source switching” button to open or close the light source switching menu, where the operator can select the light source angle 45°, 18°, and direct (80°), and the light source band, i.e., 254 nm, 365 nm, 445 nm, 485 nm, 520 nm, 580 nm, 620 nm, 750 nm, 850 nm, and 940 nm. The light source band spreads from the Ultraviolet sub-band C (UV-C) up to the near-infrared (NIR or IR-A), as illustrated in Fig. 9, while 254–940 nm is the band range used to acquire the images, and the marking wavelength is the center wavelength of the optional filter (the bandwidth is 30 nm) during imaging.

**Figure 10:**
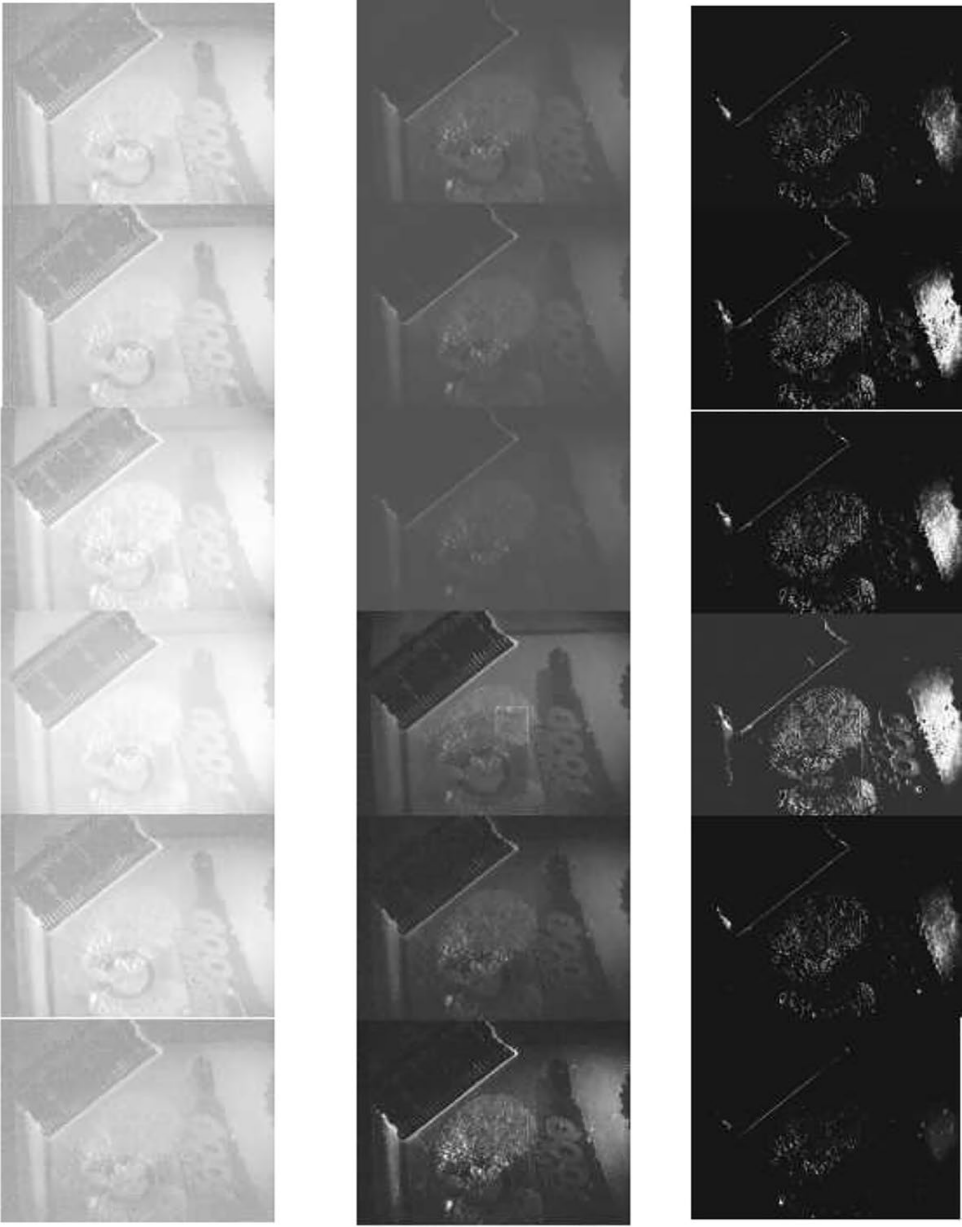
Example of three image sets using one light distribution angle.

**Figure 11:**
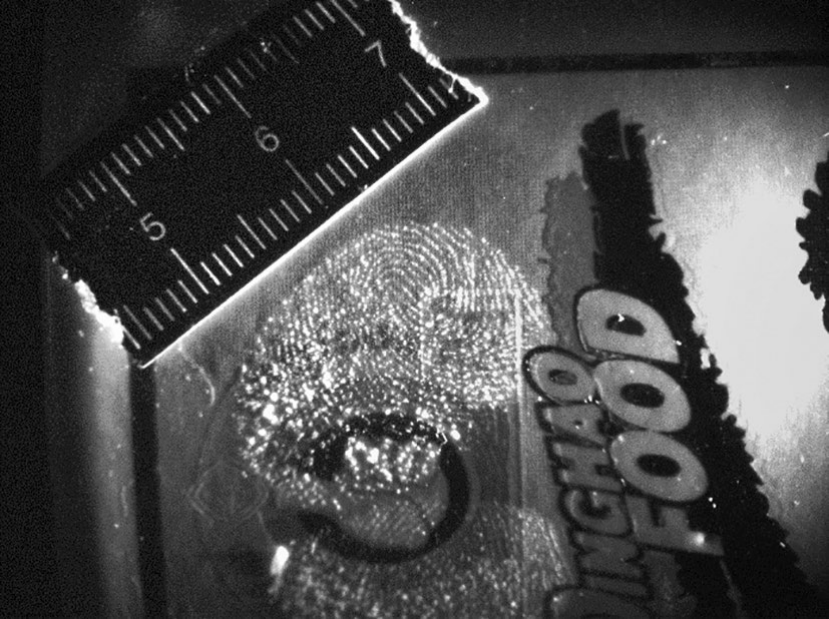
The resultant image from the case of Fig. 10.

Given the device’s operations mentioned above, using it is trivial. The operator has to press the one-click control panel to start the control system, and the device starts to operate from the reflection mode. Initially, the system employs the light distribution angle 1 and uses the six light source modules in the light distribution angle 1 to intelligently acquire the photograph. When the sixth light source module in the light distribution angle 3 activates, three image groups containing six images per group, so 18 in total, are formed, as illustrated in Fig. 10. Then, the control system automatically switches to the fluorescence mode equipped with six groups of filters. Hence, the control system automatically switches to the fluorescence mode equipped with six groups of filters and performs intelligent shooting in sequence, starting from (filter 1—light distribution angle 1—light source module 1) until (filter 6—light distribution angle 3—light source module 6), forming six groups of images (18 images per group, thus 96 images in total). The images are stored in the device’s storage module together with the images acquired in the previous reflection mode. Ultimately, a set of physical evidence images is formed (114 images in total). Once the system completes imaging (filter 6—light distribution angle 3—light source module 6), the control system starts the image processing module that automatically performs feature extraction and registration, and image optimization and fusion in the entire physical evidence image set. The processing utilizes the preset algorithm to finally form the resulting image displayed on the high-definition LCD touch screen of the one-click control panel. Figure 11 depicts the resulting image of the acquired images of Fig. 9. According to the test report of the Shanghai Institute of quality inspection and technical research, the time to generate the final image is less than 60 s, while the field tests by three industry experts require 38 s.

The evaluation and the experimental setup were based on field trials (TRL 6) conducted by an independent third party during real crime scene evidence collection. We intentionally adopted such an evaluation strategy to challenge our device in an uncontrolled and unknown environment under real crime scene evidence collection conditions. Although the operator was untrained, our easy-to-use one-click device achieved evidence collection (fingerprints in our case) exceptionally well (see Fig. 11). Nevertheless, given the opportunity for real field testing, our evaluation was constrained to a qualitative evaluation by visually inspecting the output image. Although a quantitative evaluation would be highly appealing, the uncontrolled field-testing scenario based on a real crime scene evidence collection and the confidentiality of the evidence did not allow us to perform such an evaluation.

It should be noted that this paper presents a ready-to-use one-click device where the hardware and software components are tightly coupled and fine-tuned, e.g., software, sensor, and local lighting conditions (at the sensor level). Therefore, simply substituting the visual sensor with an infrared or a High Dynamic Range (HDR) is another device requiring various modifications and in-depth setups (software and hardware). Therefore, we adopt the experimental strategy of related works on crime scene devices^18,19^ and introduce only the originally designed device rather than expanding on other alternative variations.

## Conclusion

Although considerable progress has been achieved in forensic scene optical imaging, the large variety of optical imaging methods and complicated operation procedures significantly affects the quality of the collected evidence due to the personnel’s skills. Consequently, by studying the optical properties of biological evidence and its carriers, learning the mechanism and technology of various light source bands to display biological evidence, and integrating multiple light sources and image processing techniques, this project studies multi-mode light source integration and light-guiding technologies. Moreover, by exploiting this knowledge, this paper develops a “one-click device for rapid visualization and extraction of latent evidence at the scene”, solving the problem of rapid search and discovery of potential biological evidence. Through the proposed one-click device, our system acquires photographs with multispectral and multi-angle lights as physical evidence, whose geometric registration, feature extraction, optimization, and fusion are completed in the background before the resultant image is displayed. The device ensures high-standard, high-efficiency, and high-quality scene forensics, improves evidence extraction’s scientific and technological contents, and simplifies the forensics procedures. Furthermore, using such a one-click system rapidly visualizes and extracts the potential traces at the scene while avoiding complicated operation procedures, eliminating the effect of different personnel’s technical skills on the quality of the collected evidence, and meeting the requirement for rapid disposal of crime scene evidence.

In summary, the “one-click device developed for rapid visualization and extraction of latent evidence at the scene” meets the current design requirements and is easy to carry, which is conducive to improving the discovery and extraction rate of on-site inspection for potential traces. At the same time, the device reduces the probability of losing a trace of the physical evidence due to the personnel’s experience, improves the ability of physical evidence extraction, and provides technical support for investigation and case solving. Spurred by the field-test trial results, the project has certain market competitiveness, promotion, and application prospects.

## Data Availability

The datasets used and/or analysed during the current study available from the corresponding author on reasonable request.
